# Concurrent Mpox and HSV-1 Proctitis in a Young Male With AIDS: A Case Report of Treatment Failure

**DOI:** 10.1155/crdi/6338218

**Published:** 2025-07-04

**Authors:** Mikhail Sukhoroslov, Fouad Kaddour-Hocine, Muhammad Hammad Ashraf, Navya Mandalapu, Shivani Bansal, Matthew Peachey

**Affiliations:** Department of Medicine, BronxCare Health System, Bronx, New York, USA

## Abstract

Managing the mpox in patients with advanced HIV infection and coinfections poses significant challenges. This report discusses a young male with advanced HIV (CD4 count 28) and severe concurrent mpox and HSV-1 proctitis. Despite initial treatment with oral tecovirimat, acyclovir, and antiretrovirals, the patient's condition worsened, requiring readmission. The patient received intravenous tecovirimat, vaccinia immune globulin, and brincidofovir. Sigmoidoscopy revealed extensive rectal and sigmoid lesions that necessitated prolonged hospitalization and pain management. This case emphasizes the complexity of treating severe coinfections in immunocompromised patients, highlighting the need for a multidisciplinary approach and consideration of alternative therapies when standard treatment fails.

## 1. Introduction

Mpox is a zoonotic viral disease that can cause significant morbidity, particularly in immunocompromised individuals. The management of mpox in patients with advanced HIV/AIDS presents unique challenges, particularly in cases of coinfection with other sexually transmitted infections, such as HSV-1. This case report details the clinical course and management strategies of a young male with advanced HIV infection, severe concurrent mpox, and HSV-1 proctitis.

## 2. Case Description

**Figure 1:**
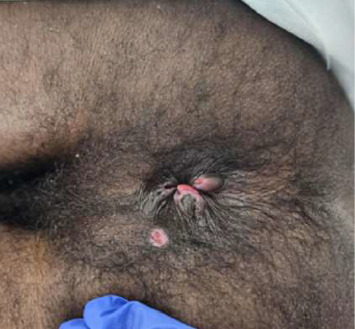
Two external anal raised lesions with ulcerated centers and a questionable ulcer in the 6 o' clock region of the anus.

The patient initially presented to the emergency department (ED) with complaints of severe rectal pain, mucus, and blood in the stool. Patient endorsed that he is engaged in receptive intercourse without consistent condom use. The patient had a known history of HIV infection with inconsistent use of HAART, missing approximately three doses per week. Skin examination revealed three nontender circular-raised lesions with ulcerated centers located on his right hand and left nostril that developed a few days ago. Rectal examination revealed two external anal nontender raised lesions with ulcerated centers and a questionable ulcer in the 6 o' clock region of the anus (Figure 1). Mpox anorectal, throat and nostril swabs, *C. trachomatis* and *N. gonorrhoeae* anorectal, throat swabs, and urine antigen were obtained, 1 dose of IM Ceftriaxone was administered, and the patient was discharged from the ED on oral doxycycline, oxycodone, and lidocaine cream.

After 2 days, the patient presented to the ED again with severe rectal pain that made him avoid liquids and food. On the day of admission, the patient showed clinical signs of dehydration and serology confirmed prerenal AKI. In addition, the patient was found to be COVID-19 positive. HIV-1 viral load was 525k copies (log 5.72), with an absolute CD4 count of 28. HCV antibody was nonreactive, and Hepatitis B core antibody was reactive.

Mpox came back positive in all of the obtained swabs (anorectal, throat, and nostril). *C. trachomatis* and *N. gonorrhoeae* were positive in throat swab and negative in anorectal swab and urine antigen test. HSV-1 and HSV-2 rectal swabs were obtained and later HSV-1 came back positive and HSV-2 negative. The patient was started on PO tecovirimat, doxycycline, sulfamethoxazole and trimethoprim, bictegravir, emtricitabine and tenofovir alafenamide, and IV acyclovir. Nutritionist was involved in the case for providing high-fat meals to increase tecovirimat absorption.

After 7 days of treatment, the patient was discharged on PO tecovirimat, valacyclovir, sulfamethoxazole and trimethoprim, and bictegravir, emtricitabine, and tenofovir alafenamide. On the following day, the patient returned with unbearable rectal pain.

Also, it was noted that the patient developed a new posterior scalp lesion. The lack of clinical improvement with PO tecovirimat was attributed to poor oral intake, and the patient was started on tecovirimat IV. In addition, he received two doses of vaccinia IVIG and brincidofovir PO.

**Figure 2:**
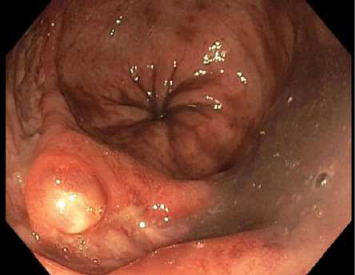
Nodular lesion in the sigmoid colon on sigmoidoscopy.

**Figure 3:**
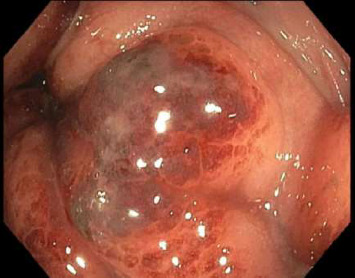
Ulcerative lesion in the rectum on sigmoidoscopy.

Sigmoidoscopy revealed nodular lesions that were extending into the distal sigmoid colon approximately 15 cm proximal to the anus (Figure 2) and one ulcerative lesion in the rectum (Figure 3). No specimens were collected because of patient's discomfort and hypotension, necessitating sigmoidoscope withdrawal.

This admission lasted for 4 weeks, and the patient was treated with tecovirimat IV for 7 days and then PO for 10 days. Swabs taken on the 34^th^ day after the onset of rectal pain, the 5th day of the second round of PO tecovirimat, were still positive for mpox. Serum tecovirimat levels and resistance testing were not sent as these testings are not available in the lab affiliated with our hospital. For HSV infection, acyclovir IV was administered for 15 days. On the 10^th^ day of IV acyclovir administration, the rectal swab tested negative for HSV. The summary of the clinical course is provided in Table 1.

The patient was discharged on hydromorphone, topical hydrocortisone, and lidocaine cream. On outreach after discharge, the patient reported rectal pain that was difficult to control. Subsequent evaluations or laboratory tests of the patient are unknown as the patient lost to follow-up with the scheduled appointments.

## 3. Discussion

This case highlights the complexities of managing severe coinfections in immunocompromised patients and the necessity for reconsidering therapeutic approaches.

The overlapping symptoms of mpox and HSV-1, coupled with the patient's advanced immunodepression and inability to tolerate oral intake, complicate the clinical picture. Most reported cases of mpox proctitis describe a relatively benign clinical presentation without definite ulceration with mild pain syndrome [1, 2], which rapidly improves after starting oral tecovirimat [3, 4]. In a few reported cases, intravenous therapy was required. One report describes a case of severe proctitis with rectal mucosal ulcerations that eventually symptomatically improved after a course of oral tecovirimat [5]. In another reported case, mpox proctitis leads to rectal wall perforation, although patient improved without surgical intervention [6]. One case report describes presentations of severe rectal pain requiring ICU admission for patient-controlled analgesia (PCA) [7]. In all of the case reports mentioned above, the symptoms resolved after appropriate course of treatment.

In this case, the initial failure of oral therapy at first was attributed to either poor oral intake (intestinal absorption of tecovirimat requires administration of high-fat meals) or resistance to oral treatment. However, despite treatment with IV tecovirimat, repeated doses of vaccinia IVIG, and brincidofovir that were administered according to the CDC's released targeted clinical guidelines for severe mpox [8], the patient still remained symptomatic.

Mpox PCR testing was still positive on the 34^th^ day of symptom onset after most of the tecovirimat treatment course. In a prospective cohort study, the median time from symptom onset to viral clearance was 16 days (95% CI: 13–23) [9]. The maximum reported length of mpox DNA positivity in saliva was 76 days [10].

In the present case, persistence of symptoms along with the absence of viral clearance on the rectal swab after a prolonged course of extensive treatment raises suspicion for failure of standard treatment due to potential issues with absorption, resistance, or inadequate host response. It highlights the importance of early recognition of mpox treatment failure, especially in immunocompromised patients. This case reinforces the importance of a multidisciplinary approach and timely access to advanced therapeutics for vulnerable populations. Delays in appropriate treatment escalation may contribute to prolonged morbidity and increased transmission risk.

## Figures and Tables

**Table 1:** Summary of the clinical course.

Day	Clinical event/finding	Treatment initiated	Laboratory/diagnostic results
Day 0	Initial ED visit with rectal pain, mucus, blood in stool, skin/mucosal lesions	1 dose of IM ceftriaxone; discharged on PO doxycycline oxycodone, lidocaine cream	Mpox anorectal, nostril, throat swabs; *C. trachomatis* and *N. gonorrhoeae* urine, rectal, and throat swabs collected
Day 2	Return to the ED with worsening rectal pain, poor oral intake, dehydration	Hospital admission	HIV VL: 525,000 copies/mL; CD4: 28; COVID+, HBcAb+, HCV Ab-
Day 3	—	PO tecovirimat, doxycycline, SMX/TMP, HAART, IV acyclovir, IM ceftriaxone	Confirmed mpox (anorectal, nostril, throat); *C. trachomatis*, *N. gonorrhoeae* throat swabs +, anorectal and urine antigen negative; HSV-1 anorectal swab +, HSV-2 anorectal swab negative
Day 10	Discharged on PO tecovirimat, SMX/TMP, HAART, valacyclovir	—	—
Day 11	Return to the ED with worsening pain, new scalp lesion	IV tecovirimat initiated; vaccinia IVIG x2; IV acyclovir, PO brincidofovir started	—
Day 13	Sigmoidoscopy: nodular and ulcerative lesions, scope withdrawn early	—	No biopsy due to patient instability and severe pain
Days 11–18	IV tecovirimat course (7 days)	Continued IV acyclovir	—
Days 19–28	PO tecovirimat resumed (10 days)	—	—
Day 20	—	—	HSV-1 anorectal swab is negative
Day 23	—	—	Mpox rectal swab still +
Day 36	Discharged with hydromorphone, hydrocortisone cream, lidocaine	—	—

## Data Availability

The data that support the findings of this study are available on request from the corresponding author. The data are not publicly available due to privacy or ethical restrictions.
